# Isotopic Evidence for Disrupted Copper Metabolism in Amyotrophic Lateral Sclerosis

**DOI:** 10.1016/j.isci.2018.07.023

**Published:** 2018-08-01

**Authors:** Lucie Sauzéat, Emilien Bernard, Armand Perret-Liaudet, Isabelle Quadrio, Alain Vighetto, Pierre Krolak-Salmon, Emmanuel Broussolle, Pascal Leblanc, Vincent Balter

**Affiliations:** 1 Université de Lyon, ENS de Lyon, CNRS, LGL-TPE, 69007 Lyon, France; 2 Hospices Civils de Lyon, Hôpital Neurologique Pierre Wertheimer, Centre de Ressources et de Compétence SLA de Lyon, Service de Neurologie C, Bron, France; 3 Université de Lyon, CNRS UMR5292, INSERM U1028, BioRan, Lyon, France; 4 Hospices Civils de Lyon, Neurobiology Laboratory, Biochemistry and Molecular Biology Department, Lyon, France; 5 Service Neurocognition et Neuroophtalmologie, Hôpital Neurologique, 59 Boulevard Pinel, 69677 Bron Cedex, France; 6 Centre Mémoire Ressources Recherche de Lyon, Hospices Civils de Lyon, Hôpital des Charpennes, Villeurbanne, France; 7 Université de Lyon, Faculté de Médecine Lyon Sud Charles Mérieux, Institut des Sciences Cognitives Marc Jeannerod, CNRS, UMR 5229, Lyon, France; 8 Institut NeuroMyoGène, CNRS UMR5310, INSERM U1217, Faculté de Médecine Rockefeller, Université Claude Bernard Lyon I, 8 Avenue Rockefeller, 69373 Lyon Cedex 08, France; 9 Université Lyon 1, Hospices Civils de Lyon, Centre de Recherche en Neurosciences de Lyon, équipe IMPACT, Lyon, France

**Keywords:** Nuclear Medicine, Isotope Chemistry, Neuroscience, Clinical Neuroscience

## Abstract

Redox-active metals are thought to be implicated in neurodegenerative diseases including amyotrophic lateral sclerosis (ALS). To address this point, we measured the concentrations of 12 elements and, for the first time, the stable isotope compositions of copper (redox-active) and zinc (redox-inactive) in human cerebrospinal fluids of 31 patients with ALS, 11 age-matched controls (CTRL), and 14 patients with Alzheimer disease. We first show that metal concentrations weakly discriminate patients with ALS from the two other groups. We then report that zinc isotopic compositions are similar in the three groups, but that patients with ALS have significantly 65copper-enriched isotopic compositions relative to CTRL and patients with AD. This result unambiguously demonstrates that copper is implicated in ALS. We suggest that this copper isotopic signature may result from abnormal protein aggregation in the brain parenchyma, and propose that isotopic analysis is a potential tool that may help unraveling the molecular mechanisms at work in ALS.

## Highlights

• Redox-active metals are implicated in ALS through oxidative stress
• Concentrations of these metals in CSFs of patients with ALS are non-specific
• Copper stable isotope composition in CSFs of patients with ALS are specific
• Isotopic balance between CSFs and brain is probably the mechanism

## Graphical Abstract

**Figure:**
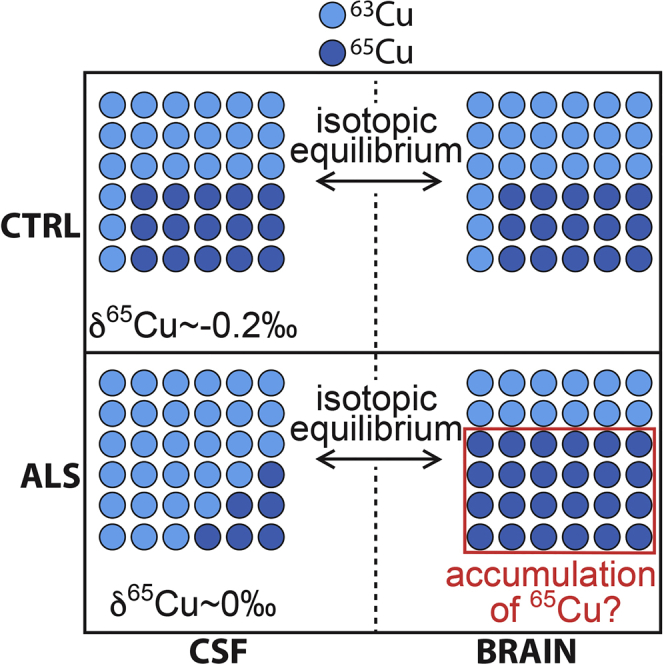

## Introduction

Amyotrophic lateral sclerosis (ALS) is one of the most harmful neurodegenerative diseases characterized by the progressive deterioration of the upper and lower motor neurons, leading to muscle weakness and atrophy, severe paralysis, and finally death typically within 3–5 years after symptom onset (Narasimhan, 2015). Currently, although genetic factors have been identified to play a role in familial ALS, which represents only 10% of the reported cases (Taylor et al., 2016), the leading mechanisms accounting for motor neuron degeneration in sporadic ALS (i.e., 90% of ALS cases) remains unknown. In addition, because of the complexity of the pathology, reliable markers (Savage, 2017) and consequently efficient treatments are still missing (Scott, 2017). Recently, biomarkers in cerebrospinal fluid (CSF) including concentrations of neurofilament light chain (Lu et al., 2015) and chemokines (Lind et al., 2016) have been identified. However, concentrations of neurofilament light chain in CSF have also been reported to be elevated in other neurological disorders such as Alzheimer disease (AD) (Zetterberg et al., 2016) and Parkinson disease (Bäckström et al., 2015). Robust and specific ALS markers are still scarce.

As for other neurodegenerative diseases, the progression of ALS is associated with the production of free radicals such as reactive oxygen species (Barnham and Bush, 2014), the production of which can be favored by the presence of free, redox-active metals like copper (Cu) (Barnham and Bush, 2014). Conversely, these metals can also be catalytic cofactors of several enzymes, like Cu/Zn superoxide dismutase (SOD1), involved in free radical detoxification by catalyzing highly toxic products (i.e., superoxide) to less dangerous species such as dioxygen and hydrogen peroxide (Valentine et al., 2005). Redox-active metals thus play a pivotal role in both the pro- and anti-oxidant homeostasis, and it is expected that dysregulation affecting these pathways will be characterized by significant elemental impairment. In mice models of familial ALS caused by mutations in SOD1, Cu has been observed to accumulate in the spinal cord (Tokuda et al., 2013). Metal dysregulations have also been observed for other redox-active metals in CSFs of patients with ALS including iron and manganese (Barnham and Bush, 2014, Roos et al., 2013). Zinc (Zn), which is not redox active, but binds to SOD1, has also been reported to be dysregulated in CSFs of patients with ALS (Hozumi et al., 2011). Comparing published results generally leads to equivocal conclusions about the usefulness of metal concentrations in CSFs to diagnose and study ALS (Figure S1).

Contrary to concentrations, stable isotope compositions may offer a more comprehensive view on biological reactions and neurological disease progression. This is due to two main reasons. First, stable isotope compositions are measured with a precision of about two orders of magnitude better than concentrations. Second, the intensity of enrichment or depletion of a metal in a given compartment is hardly predictable, whereas isotopic fractionation, i.e., the variation of the natural abundances of stable isotope ratios between coexisting compartments, can usually be quantitatively predicted by *ab initio* calculations (Albarède et al., 2016). In blood, Cu isotope compositions (δ^65^Cu) exhibit, for example, significant differences between men and women (Jaouen et al., 2012) as well as during aging (Jaouen et al., 2013). In addition, the δ^65^Cu value varies in pathological conditions such as cancer (Balter et al., 2015), Wilson disease, or liver cirrhosis (Costas-Rodriguez et al., 2016). Regarding neurodegenerative disorders, although Cu isotope compositions seem to be insensitive to the SOD1^G93A^ mutation in the brain of mouse model (Enge et al., 2017), they are highly responsive in models of prion protein knockout (PrP-KO) mice (Büchl et al., 2008, Miller et al., 2016). Zinc isotope compositions (δ^66^Zn) were also scrutinized in mouse models of neurodegenerative diseases. In PrP-KO mice, the brain δ^66^Zn values were heavier than in wild-type controls (Büchl et al., 2008), a pattern also observed in mutant mouse (APPswe/PSEN1dE9) developing Alzheimer-like disease (Moynier et al., 2017).

In the present work, to further explore the potential of elemental concentrations and isotopic compositions for clinical studies of ALS, we measured the concentration of 12 major and trace elements and the Cu and Zn isotopic compositions in the CSFs of patients with ALS (n = 31), age-matched controls (CTRL, n = 11), and patients with AD (n = 14).

## Results

Major and trace element concentrations as well as Cu and Zn isotopic compositions measured in CSFs of subjects with ALS, CTRL, and subjects with AD are all reported in Data S1.

### ALS-Related Dysregulations of Major and Trace Element Concentrations

**Figure 1:**
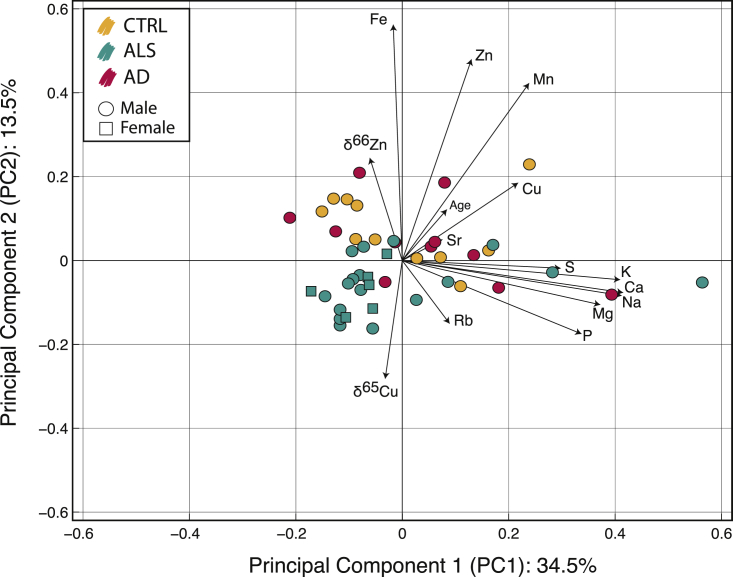
Principal Component Analysis of the Results The PCA allows the distinction of patients with ALS (green points) from CTRL subjects (yellow points) and subjects with AD (pink points). Concentrations and isotopic compositions are normalized to their SD. The two principal components (PC1 and PC2) are represented and explain ∼50% of the total variance in chemical composition. For each component, black straight lines show the loading factors (i.e., the weight of each variable on the principal components). Circles and squares points stand for male and female subjects, respectively. No significant variation is observed between the male and the female subjects within the ALS group.

To evaluate the whole pattern of variations, we used a principal component analysis (PCA) (Figure 1). The most noticeable feature is the chemical distinction between CSFs of patients with ALS and CTRL as illustrated by the y principal component axis (Figure 1). Subjects with ALS have, for example, significant lower Fe concentrations (Wilcoxon-Mann-Whitney, p value = 2.426 × 10^−3^) and Mn concentrations (Wilcoxon-Mann-Whitney, p value = 2.206 × 10^−3^) (Figure 1) but higher Rb concentrations (Wilcoxon-Mann-Whitney, p value = 1.003 × 10^−2^) (Figure 1 and Data S1). Conversely, Zn concentrations (Wilcoxon-Mann-Whitney, p value = 0.0715) and Cu concentrations (Wilcoxon-Mann-Whitney, p value = 0.1610) do not exhibit any systematics (Figure S1), an observation that also holds for other trace and major elements such as Sr, S, P, or Na (Data S1). Although these results are in line with a couple of studies, e.g., Ostachowicz et al., 2006, Kanias and Kapaki, 1997, and Kapaki et al. (1997), opposite or equivocal results have also been reported (Figure S1 and references therein).

### ALS-Related Dysregulations of Cu-Zn Isotopic Compositions

**Figure 2:**
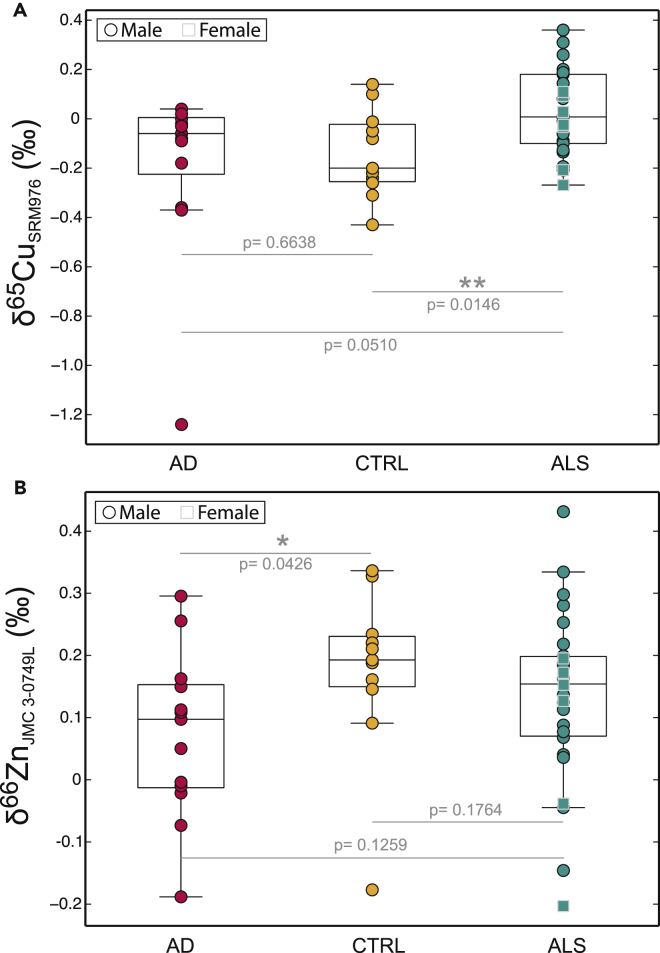
Copper (δ^65^Cu) and Zinc (δ^66^Zn) Isotopic Variability in Cerebrospinal Fluids (A and B) (A) Copper (δ^65^Cu) and (B) zinc (δ^66^Zn) isotopic compositions measured in CSFs of CTRL (yellow dots), patients with AD (red dots), and patients with ALS (green dots). Circle and square points are for male and female subjects, respectively. For each boxplot, the central mark is the median; the edges of the box are the first (i.e., 25^th^ percentiles) and the third quartiles (i.e., 75^th^ percentiles), respectively; and the points outside the boxes extend to the most extreme data points (i.e., not considered outliers). Significance level (i.e., p value) was determined using non-parametric “two-sided,” Wilcoxon-Mann-Whitney U tests. **p < 0.005, *p < 0.05.

Regarding the Cu and Zn isotopic compositions, Cu isotopic ratios in ALS (median δ^65^Cu_ALS_ = 0.01‰, 25^th^ percentile = −0.10‰; 75^th^ percentile = 0.18‰) are significantly heavier (Wilcoxon-Mann-Whitney, p value = 0.0146) than CTRL (median δ^65^Cu_CTRL_ = −0.20‰, 25^th^ percentile = −0.26‰; 75^th^ percentile = −0.02‰) (Figure 2A). By contrast, no significant difference is observed between Zn isotopic compositions of ALS (median δ^66^Zn_ALS_ = 0.15‰, 25^th^ percentile = 0.07‰; 75^th^ percentile = 0.20‰) and CTRL (median δ^66^Zn_CTRL_ = 0.19‰, 25^th^ percentile = 0.15‰; 75^th^ percentile = 0.23‰) (Wilcoxon-Mann-Whitney, p value = 0.1764) (Figure 2B).

Similar observations can be made by comparing the δ^65^Cu value of patients with ALS and AD (median δ^65^Cu_AD_ = −0.06‰, 25^th^ percentile = −0.23‰; 75^th^ percentile = 0.01‰), patients with AD being chemically indistinguishable from CTRL subjects (Wilcoxon-Mann-Whitney, p value = 0.6638) (Figure 2A). However, subjects with AD tend to have a slightly lower δ^66^Zn value (median δ^66^Zn = 0.10‰, 25^th^ percentile = −0.01‰; 75^th^ percentile = 0.15‰) than CTRL subjects (Wilcoxon-Mann-Whitney, p value = 0.0426).

### Effect of Biological and Clinical Parameters on Elemental Concentrations and Isotopic Compositions

Biological (gender and age at sample collection) and clinical (localization of first symptoms, Awaji criteria, ALSFRS-R score, delay between sampling date and first visible symptoms) parameters are given in Table S1. Focusing on the ALS and CTRL groups, neither clinical parameters nor biological parameters show significant association with elemental concentrations and Cu-Zn isotopic compositions. An illustration is the similar δ^65^Cu values observed between male and female subjects in the ALS group (median δ^65^Cu_ALS-female_ = −0.02‰, 25^th^ percentile = −0.16‰, 75^th^ percentile = 0.07‰; median δ^65^Cu_ALS-male_ = +0.01‰, 25^th^ percentile = −0.10‰, 75^th^ percentile = 0.20‰) (Wilcoxon-Mann-Whitney, p value = 0.2721) as well as between patients with ALS characterized by different site at onset (i.e., lower limbs, ML; upper limbs, MS; bulbar, bulb) (median δ^65^Cu_ALS-MI_ = 0.08‰, 25^th^ percentile = 0.01‰, 75^th^ percentile = 0.14‰; median δ^65^Cu_ALS-MS_ = −0.06‰, 25^th^ percentile = −0.15‰, 75^th^ percentile = 0.07‰; and median δ^65^Cu_ALS-bulb_ = −0.03‰, 25^th^ percentile = −0.13‰, 75^th^ percentile = 0.18‰) (Table S1).

## Discussion

Disruption of the homeostasis of metals such as Cu and Zn is a key feature of neurodegenerative diseases, including ALS, leading to multiple abnormalities in the CSF, brain, and spinal cord, where they are inappropriately redistributed (Barnham and Bush, 2014). The changes in the distribution of metals seem to be controlled by their sequestration within misfolded protein aggregates as shown, for example, in amyloid-β (Aβ) plaques within the AD brain (Pithadia and Lim, 2012). In ALS, misfolded protein aggregates including TAR DNA-binding protein 43 (TDP-43), fused in sarcoma (FUS) (Pokrishevsky et al., 2012), and SOD1 (Valentine et al., 2005) have been reported to be present in the brain. Described as the main pathological hallmark of ALS, these proteins, like Aβ for AD, may concentrate metals. The local accumulation is likely to deplete the surrounding environment, ultimately scavenging the CSF burden, leading to decreased CSF concentrations. However, no change in elemental concentrations in patients with ALS, or AD, compared with CTRL, is observed in the present study, and more generally in the literature (Figure S1 and reference therein). The absence of any systematics may result from the natural wide range of metal concentrations in human fluids (e.g., Iyengar and Wolttiez, 1988). A more robust pattern of variations of metal concentrations is probably specific to each patient and would be more accessible through analysis of cohorts. The absence of any systematics may also result from exogenous contamination. Collection, storage, and preparation of CSFs can be sensitive to trace element contamination (Garçon et al., 2017). CSFs are made of 99% water and have very low metal contents; hence the risk of contamination is high. In this study, we ensure the absence of external contamination by quantifying the chemical content that may be released by sampling procedures and storage in tubes as well as dropper-type pipettes used to collect and conserve CSF. A complete description of the procedure is given in Transparent Methods. We also took care of low-acid blanks before and after each column chemistry, and we only used vinyl gloves as suggested by Garçon et al. (2017).

One advantage of using isotopic composition over concentration for a given element is that isotopic fractionation is not dependent on the amount of the element. Isotopic compositions are thus theoretically more reliable biomarkers than concentrations. Here, we found that CSFs of patients with ALS have significantly heavier Cu isotopic composition compared with age-matched CTRL (Wilcoxon-Mann-Whitney, p value = 0.0146) and also tend to be different from those of patients with AD by having slightly heavier δ^65^Cu (Wilcoxon-Mann-Whitney, p value = 0.0510) (Figure 2A), whereas again no distinction is observed for Cu concentration (Figure S1). Using receiver operating characteristic (ROC) analysis, we determined a δ^65^Cu cutoff value of −0.05‰ with a sensitivity and a specificity of 73% and 65% respectively, and an accuracy of 76% (Figure S2). This is a modest score compared, for instance, with the results obtained by Pasinetti et al. (2006), with a sensitivity, specificity, and accuracy of 91%, 97%, and 95%, respectively, using three protein concentrations in CSF. Noteworthy is the specificity of the CSF δ^65^Cu values of ALS as illustrated by the absence of significant δ^65^Cu difference between subjects with AD and CTRL subjects (Wilcoxon-Mann-Whitney, p value = 0.6638) (Figure 2A). Further additional studies and evidence of the mechanisms at work are undoubtedly needed to improve the present results achieved by the ROC test.

Recently, Moynier et al. (2017) showed that brains of mice with AD have higher δ^66^Zn value than wild-type mice and suggested that this isotopic enrichment may result from the formation of Aβ plaques in the brain parenchyma binding preferentially heavier Zn isotopes. Following this assumption, if the binding of a metal in the protein aggregates of the brain is associated with an isotopic fractionation, this must be balanced in the CSF, the brain and CSF being two complementary reservoirs in a closed system, the CNS. At first glance, our results on patients with AD support the hypothesis of an isotopic equilibrium between brain and CSF, because patients with AD exhibit CSF depleted in heavier Zn isotopes relative to CTRL subjects. However, this requires further analysis of CSF of patients with AD and CTRL subjects, which is beyond the scope of the present work. Focusing on ALS, a similar reasoning can be proposed with aggregation of SOD1 in the brain associated with a Cu isotopic fractionation. Direct evidence could be obtained by measuring the Cu isotope composition of aggregates, but here we can test if the assumption of an isotopic equilibrium between brain and CSF resists mass conservation laws. The concentration of Cu in brain varies between 3 and 5 μg/g (Scheiber et al., 2014). The brain volume ranges from 1,300 to 1,500 mL leading to a brain Cu burden (M_B_) of 3.9–7.5 mg. Regarding CSF, the concentration of Cu ranges from 0.02 to 0.2 μg/mL (Table S1; Iyengar and Wolttiez, 1988) and, with a volume of 150–250 mL, this gives a total Cu mass (M_CSF_) of 3–5 μg. As a first approximation, the CNS can be considered as a closed system at steady state and, δ^65^Cu_B_ and δ^65^Cu_CSF_ being the Cu isotopic composition in brain and CSF, respectively, one can write that:

M_B_. δ^65^Cu_B_ ⇔ M_CSF_. δ^65^Cu_CSF_

If the isotopic composition of Cu varies in one compartment by a factor Δ, this must be balanced in the other one such that:

M_B_. Δδ^65^Cu_B_ = M_CSF_. Δδ^65^Cu_CSF_

**Figure 3:**
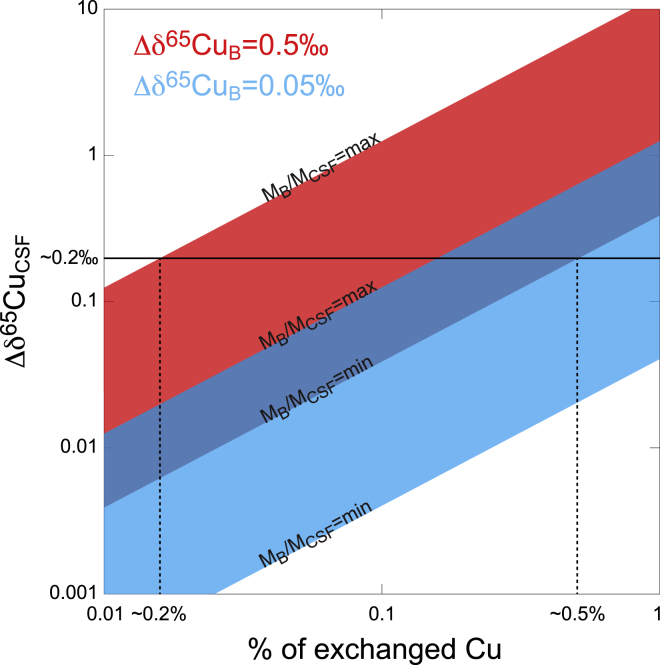
Cu Isotope Mass Balance between CSF and Brain Calculation of the Cu isotopic offset in the CSF (Δδ^65^Cu_CSF_) that results from the incorporation of Cu in the brain (% of exchanged Cu) associated with an isotopic fractionation Δδ^65^Cu_B_. The calculations are made with two values of Δδ^65^Cu_B_ (0.5‰ in red and 0.05‰ in blue) and two extreme values of the M_B_/M_CSF_ ratio. The results thus define two areas in red and blue that overlap giving the dark blue area. The results discussed in the text corresponding to some proportions of exchanged Cu for a Δδ^65^Cu_CSF_ value of 0.2‰, which represents the difference between CTRL and ALS δ^65^Cu mean values, are illustrated.

It is thus possible to calculate the Cu isotopic offset in the CSF (Δδ^65^Cu_CSF_) that would result from the incorporation of Cu in the brain with an isotopic fractionation Δδ^65^Cu_B_. The assumption that the CNS is a closed system is made for the sake of simplicity for purposes of mass balance calculations. Physiologically, metals and other molecules are supplied in the CNS by blood, but classically stay there and accumulate with time. The inward flux of Cu in the CNS by time unit is unknown but should be very small compared with the mass of the CNS, considering that the Cu amount in blood (∼5 μg) is three orders of magnitude less than that in CNS. Results of the calculations are illustrated by Figure 3. Intuitively, because there are between 78 and 2,500 more Cu in the brain than in the CSF, the Δδ^65^Cu_B_ value must be very small. Indeed, when the highest M_B_/M_CSF_ ratio is considered (2,500), a contribution of only 0.01% of pathological Cu associated with a Δδ^65^Cu_B_ value of 0.5‰ can trigger a Δδ^65^Cu_CSF_ offset of almost 0.2‰, which is the observed Cu isotopic difference between ALS and CTRL. An identical Δδ^65^Cu_CSF_ offset is obtained with a contribution of pathological Cu of 0.5% when the lowest M_B_/M_CSF_ ratio of 78 is considered. Altogether, these results show that minute proportions of aggregates formation associated with relevant Cu isotopic fractionation can likely explain the observed differences between the CSFs δ^65^Cu values of CTRL and patients with ALS. Whatever the proportion of formed aggregates, the increase of the ALS δ^65^Cu values implies for mass balance requirement that ^63^Cu preferentially binds in aggregates. Measuring the Cu isotopic composition of protein aggregates and normal adjacent areas in autopsies of brains of patients with ALS, and/or in brains of ALS animal model like the SOD1^G93A^ mice, can be the aim of future experiments to challenge this hypothesis.

Another way to explain the specific Cu isotopic composition of ALS would involve a differential Cu isotope fractionation between detergent-soluble SOD1 and aggregated, insoluble, SOD1. In mutant (G37R and G93A) SOD1 proteins from SOD1-ALS transgenic mice spinal cords, Lelie et al. (2011) found that aggregated, insoluble, SOD1 is metal depleted, whereas soluble SOD1 is highly Cu metallated. Exchange of Cu between soluble and aggregated SOD1 is unlikely because soluble SOD1 is thought to be highly stable. Lelie et al. (2011) also argued that aggregation occurs when SOD1 is in its immature, unmodified, apo form (Shaw et al., 2008). All these results suggest that the aggregation of SOD1 leads to the over-metallation of soluble SOD1. The abnormal Cu metallation of soluble SOD1 can be accompanied by different isotopic fractionation from normal conditions, which can explain the present results. Measurement of the Cu isotope composition of the soluble and insoluble fractions of SOD1-ALS transgenic mice spinal cords would help to confirm this hypothesis.

To conclude, our results, i.e., the first to report Cu and Zn isotopic compositions in CSFs of patients with ALS and AD and CTRL subjects, demonstrate that Cu isotopic measurements in CSF may offer a more comprehensive view of the ALS metallome than elemental concentrations and may potentially reinforce any diagnosis. Similarly, the lighter δ^66^Zn value observed in the CSF of patients with AD compared with that of CTRL subjects may also offer promising information regarding Zn dyshomeostasis in AD. Increasing the number of Cu and Zn isotopic measurements in CSFs of ALS and AD, respectively, as well as in other neurodegenerative pathologies, such as Parkinson or Huntington diseases, would undoubtedly challenge the proposition that δ^65^Cu and δ^66^Zn values in CSF are probably future candidate biomarkers of neurodegenerative diseases.

## Methods

All methods can be found in the accompanying Transparent Methods supplemental file.

## Supporting Citations

Costa et al., 2012, del Campo et al., 2012, Ihara et al., 2013, Jaouen et al., 2016, Kapaki et al., 1989, Maréchal et al., 1999, McKhann et al., 2011.

## Supplemental Information

Document S1. Transparent Methods, Figures S1–S3, and Table S1

Data S1. Trace and Major Element Concentrations and Cu and Zn Isotopic Compositions Measured in Cerebrospinal Fluids, Related to Figure 1

Data S2 Major and Trace Element Concentrations (ppm) and Cu-Zn Isotopic Compositions (‰) of In-House and Certified Standards Measured in this Study, Related to Figure 1
