# Supplementary material for: Isotopic Evidence for Disrupted Copper Metabolism in Amyotrophic Lateral Sclerosis
Source: iScience. 2018 Aug 1;6:264–71. doi: 10.1016/j.isci.2018.07.023 (PMC6137708; doi:10.1016/j.isci.2018.07.023)
Supplement: Document S1. Transparent Methods, Figures S1–S3, and Table S1 [file mmc1.pdf]

ISCI, Volume *6*

Supplemental Information

Isotopic Evidence for Disrupted Copper Metabolism in Amyotrophic Lateral Sclerosis

Lucie Sauzéat, Emilien Bernard, Armand Perret-Liaudet, Isabelle Quadrio, Alain Vighetto, Pierre Krolak-Salmon, Emmanuel Broussolle, Pascal Leblanc, and Vincent Balter

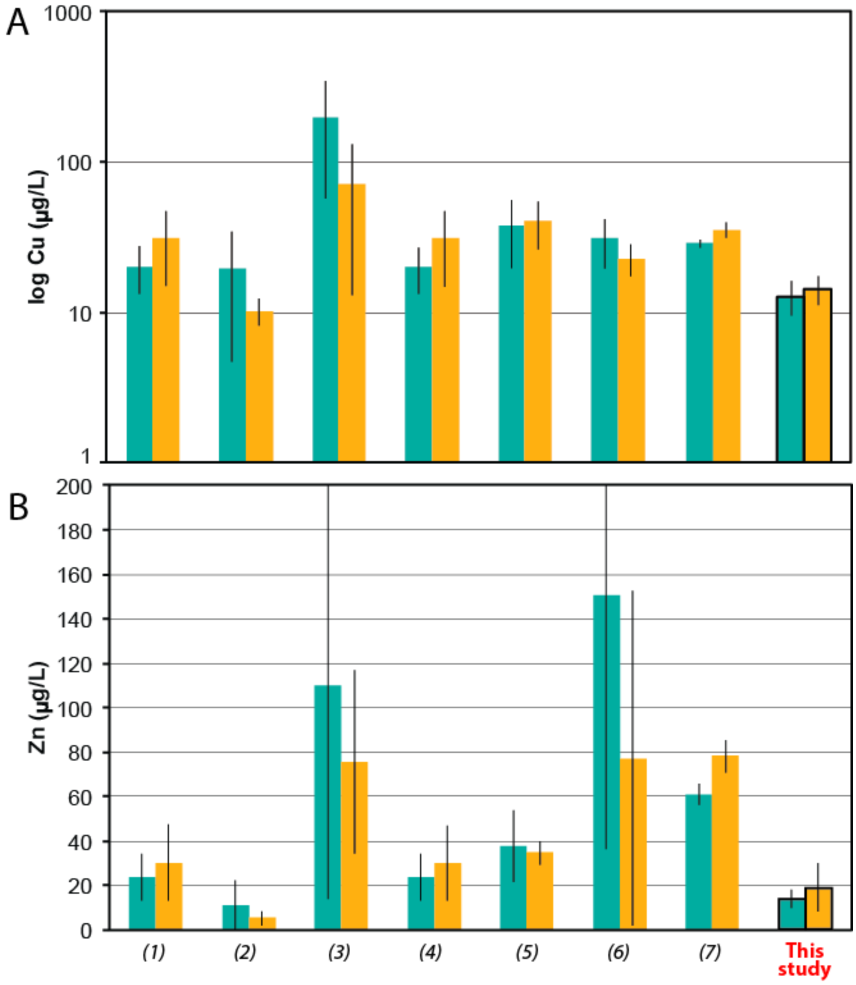

Supplementary Figure S1: Elemental concentrations variability in CSF of ALS and CTRL subjects, related to Figure 1. Compilation of a) Cu and b) Zn concentrations of cerebrospinal fluids (CSFs) in both control (yellow) and amyotrophic lateral sclerosis subjects (green), from the literature and the present study. Error bars represent two standard deviations of the mean. Cu diagram is represented with a logarithm scale. Data from the present study are labeled in red, and literature data, in italic from 1 to 7, are for Kapaki et al. (1997), Hozumi et al. (2011), Ihara et al. (2013), Kanias et al. (1997), Kapaki et al. (1989), Roos et al. (2013), Ostachowicz et al. (2006), respectively.

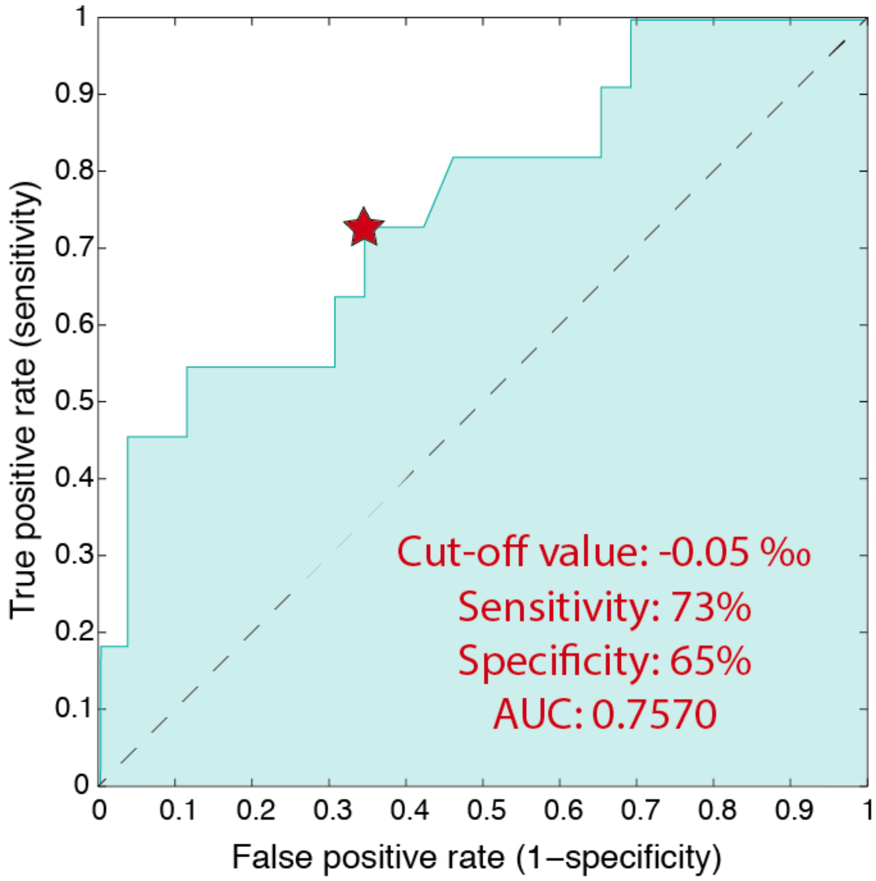

Supplementary Figure S2: Receiver Operating Curve (ROC), related to Figure 2. ROC analysis of the $\delta^{65}Cu$ values in CSF of ALS patients compared to that of CTRL subjects.

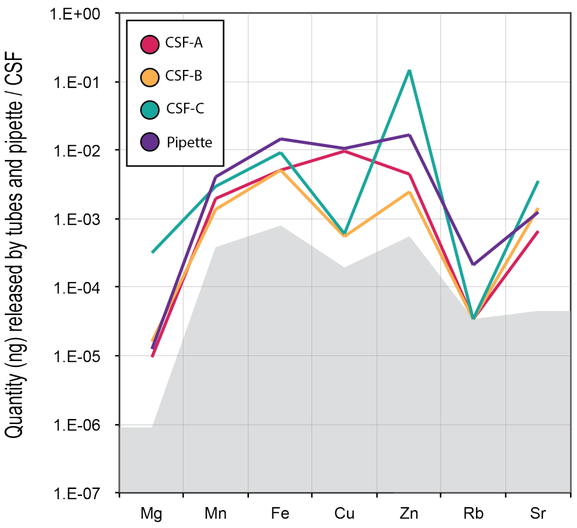

Supplementary Figure S3: Estimation of blank levels, related to Figure 2. Amount of trace element released by sampling and storage tubes as well as dropper-like pipette normalized to the amount of elements initially present in 2mL of cerebrospinal fluids (CSFs) (i.e. the average volume analyzed in this study). The grey field represents values below the limit of detection (DL) defined following IUPAC guideline (i.e. $DL_i = xb_i + k*sb_i$ where k=3, xb_i and sb_i are respectively the mean and the standard deviation of the number of counts measured in blanks). When the amount detected in the solution was lower the limit of detection, data were represented as equal to the detection limit.

Supplementary Table S1: General information and patients' clinical records, related to Figure 1

Sample Name	Gender	Age (years) at sample collection	Localization of first symptoms *	Awaji criteria	ALSFRS-R **	Time (months) between sampling date and first symptoms
ALS1	Male	45	MI	definite	44	7
ALS2	Male	62	MS	definite	39	6
ALS4	Male	81	MS	definite	40	6
ALS5	Male	46	bulb	definite	40	12
ALS6	Male	34	MS	definite	42	12
ALS7	Male	65	bulb	definite	44	12
ALS8	Male	86	MI	possible	37	6
ALS9	Male	68	MI	definite	45	12
ALS10	Male	73	MS	definite	37	6
ALS11	Male	64	MS	probable	28	6
ALS12	Male	60	MS	definite	33	6
ALS13	Male	60	bulb	definite	34	6
ALS14	Male	57	MI	definite	32	24
ALS 15	Male	49	bulb	definite	41	6
ALS 16	Male	50	MS	probable	39	12
ALS 17	Male	69	MI	probable	45	36
ALS 18	Male	60	bulb	definite	39	24
ALS 19	Male	49	bulb	definite	39	12
ALS 20	Male	75	MI	probable	35	12
ALS 21	Male	70	MS	definite	23	24
ALS 22	Male	76	bulb	definite	42	12
ALS 23	Male	54	bulb	definite	43	7
ALS 24	Male	44	MI	probable	46	24
ALS 25	Female	71	MS	probable	43	4
ALS 26	Female	64	bulb	definite	40	6
ALS 27	Female	63	bulb	definite	40	24
ALS 28	Female	67	MI	definite	40	5
ALS 29	Female	69	MI	probable	34	6
ALS 30	Female	64	MI	probable	40	9
ALS 31	Female	45	MS	definite	41	24
ALS 32	Female	43	MI	probable	42	6
CTRL2	Male	49				
CTRL3	Male	62				
CTRL4	Male	69				
CTRL5	Male	48				
CTRL6	Male	46				
CTRL7	Male	67			n/a	
CTRL8	Male	60				
CTRL9	Male	44				
CTRL10	Male	70				
CTRL11	Male	58				
CTRL 12	Male	79				
AD4	Male	75				
AD5	Male	78				
AD6	Male	68				
AD7	Male	68				
AD8	Male	78				
AD9	Male	63				
AD10	Male	70			n/a	
AD11	Male	59				
AD12	Male	63				
AD13	Male	71				
AD14	Male	73				
AD15	Male	73				
AD16	Male	79				
AD17	Male	78				

Footnote:
"n/a" stands for unspecified value
CTRL stands for control subjects, ALS and AD are for Amyotrophic lateral sclerosis and Alzheimer patients respectively

*Site at onset: - MI=lower limbs
- MS=upper limbs
- bulb=bulbar

**ALSFRS-R stands for the revised version of the Amyotrophic Lateral Sclerosis Rating scale

Transparent methods

Subjects and samples

In this study, all subjects were hospitalized in the Department of Neurology for diagnosis purposes, which included cerebrospinal fluid (CSF) analyses among other tests. All patients signed informed consent about the potential use of their CSF for further research purposes and an ethical approval was also delivered by the local Ethics committee of the Hospices Civils de Lyon (date of delivery: 7th October, 2016). We investigated the CSF of 31 ALS patients diagnosed using the Awaji criteria (Costa et al., 2012). They were compared to a group of 25 patients suffering from neurocognitive complaint referred to expert memory clinic linked to Lyon Center for Memory Resources and Research. These 25 patients underwent lumbar puncture using a standard procedure (del Campo et al., 2012) in the context of clinical diagnosis of neurodegenerative diseases. Cerebrospinal fluid biomarker for AD pathology (*i.e* total and phosphorylated TAU proteins and Amyloid beta 1-42 peptide) were performed blind to the clinical diagnosis in the Neurochemistry Unit of Lyon University Hospital using commercially available enzyme-linked immunosorbent assays (INNOTEST hTau-Ag, INNOTEST phosphorylated-Tau181, and INNOTEST Aβ1-42; Fujirebio Europe) according to the manufacturer's instructions. The Neurochemistry Unit participated in the Alzheimer Association Quality Control Program for these biomarkers. Diagnoses were finally proposed in the framework of multidisciplinary consultation taking into account medical history, caregivers interviews, neurologic examination, neuropsychological evaluation, brain imaging and results of cerebrospinal fluid biomarker for AD pathology. AD diagnostic was excluded in 11 of these 25 patients who were finally classified as suffering mainly from psychiatric conditions and were then considered as controls for this study. The remaining 14 patients met the diagnostic criteria of Alzheimer 's disease according to McKhann et al. (2011). Both male and female from different ages ranging from 34 to 86 years old were studied. To avoid any sample bias, we also considered diverse ALS cases *i.e.* characterized by different onset brain location (bulbar, lower and upper limbs), distinct value of Awaji criteria and ALSFRS as well as variable time of symptom onset (6 to 36 months). All the details are summarized in Supplementary Table S1.

Major and trace element concentrations

All chemical analyses were carried out in clean laminar flow hoods using double-distilled acids to avoid any exogenous contaminations. Samples were first weighted and then dissolved in a mixture of 15M HNO_3 and H_2O_2 (30%) in Savillex® beakers at 120°C for about 72h. When dissolved, major and trace element concentrations were measured in a small aliquot on an ICP-AES (iCAP 6000 Radial) and a quadrupole ICP-MS Thermo iCap-Q respectively at the Ecole Normale Supérieure (ENS) of Lyon following the method described in Garçon et al. (2017). Trace and major element concentrations are reported in ng/mL and µg/mL respectively in Supplementary Dataset 1. Briefly, the concentrations were calculated using calibration curves based on multi-elemental solutions. These solutions were also used to monitor and correct the instrumental drift over the analytical session. Oxide interference and analytical drift were also corrected using indium (In) and scandium (Sc) addition as internal standards for trace and major elements, respectively. The precision of the results was assessed by complete duplicate and re-run analyses (referred as “dup” and “bis” samples respectively in Supplementary Dataset 1) and accuracy and reproducibility were monitored by replication of certified reference materials (1577c, DORM2) and an in-house standards (OEP, FBS) measured as unknown samples (Supplementary Dataset 2). The results are generally reproducible and consistent within 10% (2sd) of previous published data (see Supplementary Dataset 2). Based on the analysis of reference standards and duplicates analyses, we therefore estimate that the measurement precision is, on average, better than 10% for both major and trace elements.

Copper and zinc isotopic compositions

Samples were purified by ion-exchange chromatography using quartz columns filled with 1.8mL of Bio-Rad AGMP-1 (100-200 mesh) anion-exchange resin. Copper and zinc were successively eluted with 20mL of HCl 7M + 0.001% H_2O_2 and 10mL of HNO_3 0.5M respectively following the procedure described by Maréchal et al. (1999). The total procedural blanks were on average 0.4 ng for Cu (n=7) and 3.0 ng for Zn (n=7), which is below the amount of element isolated from the sample and available for isotopic measurement (*i.e.* average Cu_{CSFs} and Zn_{CSFs} of 20 ng). Blank contribution in the samples is therefore higher for Zn than for Cu. To

ensure the quality of the Zn isotopic data, we quantified the impact of this exogenous contamination using the mixing equation provided by Garçon et al. (2017). Tubes and pipettes used to collect and store the CSFs cannot release significant amount that may account for exogenous contamination (see below *Effect of exogenous contamination induced by tubes and pipettes on major and trace element concentrations measured in CSFs*). Conversely, gloves contain high Zn content that may be easily mobilized during sample preparation (Garçon et al., 2017). Gloves are probably the main source of contamination. With a $\delta^{66}Zn_{gloves}$ of 0.10 ± 0.32 ‰ (2sd), we show that for Zn = 20 ng (*i.e.* the content available for isotopic measurement in the samples), no significant shift can however be induced beyond the measurement uncertainties (*i.e.* ±0.07 ‰, 2sd) (Supplementary figure 3) ensuring the reliability of our isotopic data measurements.

The isotopic compositions of Cu and Zn are measured by multi-collector inductively coupled plasma mass spectrometry (MC-ICP-MS, Nu500) in wet plasma conditions following the procedure described by Maréchal et al. (1999). On the day of analyses, Cu and Zn purified solutions are diluted in a Zn-doped solution (Zn JMC 3-0749L, Johnson Matthey Royston, UK) and a Cu-doped solution (Cu SRM 976, National Institute of Standards and Technology, Gaithersburg, MD, USA), respectively, to match the concentration of the standard mixture run between the samples (between 75 and 300 μg.L^{-1} depending on the sample). The delta values (expressed in ‰) are reported relative to the isotopic solution reference material NIST SRM 976 for Cu and JMC 3-0749L for Zn and are referred as:

$$\delta^{65}Cu = \left[\frac{\left({}^{65}Cu/{}^{63}Cu\right)_{sample}}{\left({}^{65}Cu/{}^{63}Cu\right)_{standard}} - 1\right] \times 1000 \text{ and } \delta^{66}Zn = \left[\frac{\left({}^{66}Zn/{}^{64}Zn\right)_{sample}}{\left({}^{66}Zn/{}^{64}Zn\right)_{standard}} - 1\right] \times 1000$$

Instrumental mass fractionation and temporal drift is corrected with an exponential law using the elemental-doping method and standard-sample bracketing respectively as recommended by Maréchal et al. (1999). The precision and the accuracy of Cu and Zn isotopic ratios were assessed by repeated measurements of re-run and duplicate samples and by reference materials (1577c, bovine liver) and in-house standard solutions (OEP, sheep plasma), respectively. The average $\delta^{66}Zn$ of reference materials 1577c and OEP were -0.19 ± 0.04 (2sd, n=4) and +0.64 ± 0.06 (2sd, n=2), respectively, which is in good agreement with our in-house previous average values: $\delta^{66}Zn_{1577c}$ = -0.19 ± 0.05 (2sd, n=13) and $\delta^{66}Zn_{OEP}$ = +0.72 ± 0.08 (2sd, n=15) as well as with previous published results ($\delta^{66}Zn_{1577c}$ = -0.13 ± 0.02 (2sd,

n=4), *37*) (Jaouen et al., 2016) (Supplementary Dataset 2). For $\delta^{65}Cu$, we measured +0.35 ± 0.07 (2sd, n=4) for 1577c and -1.16 ± 0.03 (2sd, n=3) for OEP which is also in agreement with our in-house reference values: $\delta^{65}Cu_{1577c}$ = +0.37 ± 0.12 (2sd, n=10) and $\delta^{65}Cu_{OEP}$ = -1.14 ± 0.09 (2sd, n=20) (Supplementary Dataset 2). Based on these results, we estimate the 2sd analytical uncertainty of our isotopic measurements at ± 0.07. Note that the long-term precision based on the repeated measurements of the pure Zn JMC 3-0749L and Cu SRM 976 solutions run every two samples are very similar to these values (± 0.05 ‰ (2s, n = 140)).

Principal component analysis

In this study, we used a correlation-based principal component analysis (PCA) to discriminate ALS patients from AD patients and age-matched CTRL as well as to quantify the effect of the ALS disease on elemental concentrations and Cu-Zn isotopic compositions. The method consists in identifying new variables called principal components (PCs), which are linear combination of the original variables and along which data variation is maximal. The variables include the chemical concentrations of 12 major and trace elements measured in cerebrospinal fluids of ALS, AD and control subjects, as well as $\delta^{65}Cu$ and $\delta^{66}Zn$ values. All data were normalized, and samples with incomplete data were excluded. PCA was implemented in MATLAB™.

Boxplot

In the present study, all the boxplot diagrams were implemented in MATLAB™ and significance level was determined using a non-parametric 'two-sided', Wilcoxon-Mann-Whitney U-test. For each boxplot, the central mark is the median, the edges of the box are the first (*i.e.* 25th percentiles) and third quartiles (*i.e.* 75th percentiles) respectively and the whiskers extend to the most extreme data points (*i.e.* not considered outliers).

ROC

Receiver Operating Curve (ROC) was used to evaluate the reliability of $\delta^{65}Cu$ values as a potential ALS diagnostic test with the aim to confirm the presence of ALS disease but also to rule out the presence of this pathology in healthy subjects. In this study, the ROC test was implemented in MATLAB™ and four distinctive

parameters were obtained: 1) the cut-off value *i.e.* the threshold value discriminating ALS form CTRL subjects; 2) the true positive rate (*i.e.* sensitivity) corresponding to the probability that a result will be higher than the cut-off value when the ALS disease is present; 3) the false positive rate (*i.e.* 1-specificity) defined as the probability that a result will be lower than the cut-off value when the ALS disease is not present; 4) The area under the curve (AUC). Optimal performance test correspond to a sensitivity and specificity of 100% and an AUC of 1.

Effect of exogenous contamination induced by tubes and pipettes on major and trace element concentrations measured in CSFs.

Cerebrospinal fluids (CSFs) are made of 99% water and have low amount of major and trace elements. To ensure the absence of external contamination in CSFs during sample collection and/or storage, we quantify the content of the most contaminable trace elements (*i.e.* Mn, Fe, Cu, Zn, Rb and Sr as well as Mg) that may be released by tubes and pipettes being directly in contact with the samples. This includes sampling (CSF-A: polypropylene, Sarstedt, 10mL, 92x15.3mm and CSF-B: polypropylene, Sarstedt, 5mL, 57x15.3mm) and storage tubes (CSF-C: polypropylene, Sarstedt, 1.5mL) as well as dropper-type pipette.

The detailed procedure consist of putting 2mL of HNO_3 0.5M + 2ppb indium (In) in the tubes, before storing them 3 weeks in a fridge. The volume of 2mL corresponds to the average volume of CSFs analyzed in this study. The duration of the test correspond to the approximate period for which CSF samples were stored in the tubes. To assess the amount of trace elements released by dropper-type pipette, we used a slightly different technique. For this test, we pour 2mL of HNO_3 0.5M + 2ppb In in clean savillex® beaker and washed the pipette three times with the solution. An aliquot of each solution is then analyzed on the Thermo iCap-Q mass spectrometer following the method described in the *Major and trace element concentrations* section.

For all the elements measured in this study, the amount released by tubes and pipette that have been in contact with low concentrated HNO_3 acid is always lower (<0.01) than the quantity present in the CSF samples (Supplementary figure S3).

Only one exception is noted for the amount of Zn released by the dropper-type pipette, but this amount remains relatively low (<0.1) compared to the amount initially present in the samples. Although we used low concentrated acid solutions, it

is important to note that chemical elements are more easily released in acidic solution than when they are in contact with non-acidic CSF samples. Therefore, our study likely provides maximized results being ten times higher that the real amounts released by tubes and pipettes when they are only filled with CSFs.

Altogether, these results show that storage and sampling tubes and dropper-type pipettes cannot induce significant exogenous contamination and bias the Mg and trace element concentrations including Fe, Cu, Zn, Mn, Rb and Sr measured in CSFs. Similar conclusion can also be drawn for Ca, K, Na, P and S, the latters being initially more concentrated in CSFs compared to trace elements.
